# Renal Involvement and Its Treatment in Pediatric Patients With Proteinase-3 Anti-Neutrophil Cytoplasmic Antibody Positive Granulomatosis With Polyangiitis: A Case Series

**DOI:** 10.7759/cureus.18197

**Published:** 2021-09-22

**Authors:** Xiaoyan Wu, Elizabeth Mancuso, Isabel Armendi, David Krasinski, Lin Liu, Shauna Tarsi, Wayne R Waz, Rabheh Abdul-Aziz, Ewa Elenberg

**Affiliations:** 1 Department of Pediatric Nephrology, State University of New York, Buffalo, USA; 2 Department of Pediatrics, State University of New York, Buffalo, USA; 3 Department of Pathology and Anatomical Sciences, State University of New York, Buffalo, USA; 4 Department of Pediatric Rheumatology, State University of New York, Buffalo, USA; 5 Department of Pediatric Nephrology, Baylor College of Medicine, Houston, USA

**Keywords:** pauci-immune anti-neutrophil cytoplasmic antibody (anca)-associated vasculitis (aav), proteinuria, hematuria, pulmonary hemorrhage, pseudotumor cerebri, proteinase-3, rituximab, therapeutic plasma exchange, cytoxan

## Abstract

We describe three pediatric patients between the ages of 10 and 14 years old who were diagnosed with granulomatosis with polyangiitis (GPA) between 2014 and 2019. Each case involves variations in presentation, symptomatology, diagnostics, and induction and maintenance therapy regimens. Patient 1 presented with significant renal involvement, hypertensive emergency, and focal alveolar hemorrhage, a rare presentation of GPA that causes up to 60% mortality.Patient 2 presented with minimal renal involvement and a diffuse petechial rash, which is the most common cutaneous presentation of GPA. Finally, patient 3 presented with significant renal involvement and later on with symptoms of idiopathic intracranial hypertension (IIH), a unique and rare presentation associated with GPA. Despite the heterogeneity of these cases, the similar therapy regimens used in each case successfully achieved induction and maintenance of disease remission, providing an evidentiary basis for these treatment regimens even in severe and unusual pediatric GPA cases.

## Introduction

Granulomatosis with polyangiitis (GPA) is a pauci-immune anti-neutrophil cytoplasmic antibody (ANCA)-associated vasculitis (AAV) characterized by necrotizing inflammation of small- and medium-sized blood vessels, resulting in a spectrum of phenotypes that range from limited local disease to systemic involvement with organ failure. In GPA, anti-neutrophil cytoplasmic antibodies (ANCA) are formed against neutrophilic proteinase-3, thus stimulating a cascade of unregulated inflammation that commonly affects the small- and medium-sized blood vessels of the kidneys and lungs. GPA is the most common AAV overall, with an incidence of 1.2 per 100,000 and on the rise [1-3]. Genetics, environment, and variations in both innate and adaptive immunity all play major roles in the pathogenesis of GPA [4,5]. The classic presentation of GPA is characterized by upper and lower respiratory tract involvement, demonstrated by chronic sinusitis, pulmonary disease, acute kidney injury (AKI), arthralgia, a leukocytoclastic, petechial or purpuric rash, and constitutional symptoms. Kidney involvement usually presents as a rapidly progressive crescentic glomerulonephritis, evident by a decline in renal function accompanied by hematuria, proteinuria, and hypertension.

Over 75% of all AAV patients initially present with rapidly progressive glomerulonephritis, with GPA typically exhibiting more severe renal disease on initial presentation than other ANCA-associated vasculitis [5]. Kidney involvement is also the most important predictor of mortality in AAV - those with estimated glomerular filtration rates (eGFRs) less than 50 mL/min have a 50% risk for death or kidney failure 5 years after diagnosis [5]. In the pediatric population, the median age of GPA onset ranges from 11.7 to 14 years [6-8]. With 83% of newly diagnosed pediatric GPA patients presenting with glomerulonephritis, this disease poses a significant risk of morbidity and mortality in a young and often otherwise healthy patient population [9]. Therefore, urgent initiation of induction therapy is integral in preventing progression to renal failure and chronic kidney disease (CKD). Proper selection of therapies is critical, as the mortality of untreated AAV can be up to 80% [10,11]. Despite renal involvement being the major determinant of disease progression, morbidity, and mortality, there are no pediatric nephrology consensus guidelines that outline the acute and chronic management of AKI or CKD in patients with GPA.

The European League Against Rheumatism (EULAR) released updated consensus guidelines for AAV management in 2016 on induction and maintenance therapies [12]. Current recommendations consist of an induction therapy initiated within three to six months after diagnosis, with the goal of counteracting the acute inflammatory process as rapidly as possible while minimizing tissue and organ damage, followed by a maintenance phase over the following 24 to 48 months, in which the principal goal is preventing disease relapse. However, these recommendations are predicated on adult literature. Only a small number of retrospective studies on GPA have been conducted in the pediatric population [13-17]. Thus, there is need for more contributory data on effective induction and maintenance therapies for GPA in pediatric patients. Even though the three cases described here share commonalities in symptomatology, with each case demonstrating renal and upper respiratory tract involvement, each patient also had a unique presentation with large differences in disease severity. Despite these differences in symptomatology, remission was achieved in each patient by using a similar induction regimen.

## Case presentation

Case 1 (patient with hypertensive emergency)

**Figure 1:**
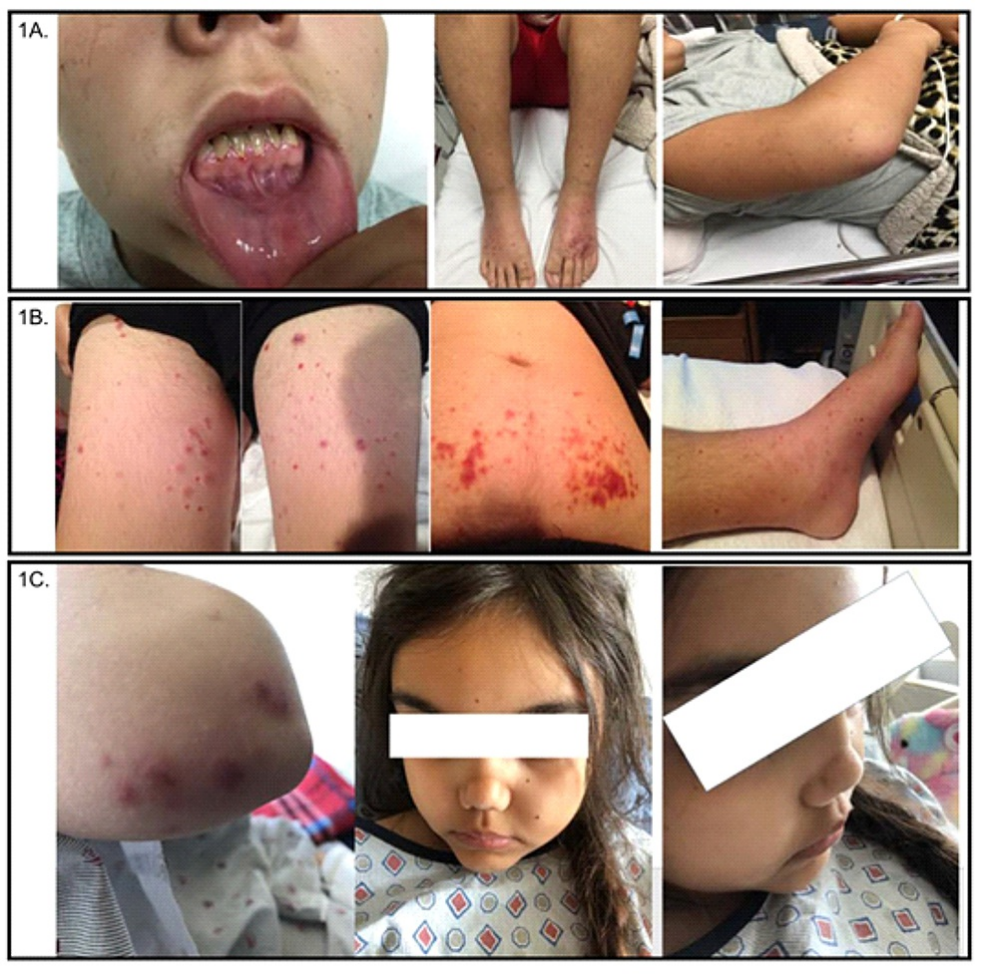
Physical examination findings on three patients. (A) The patient in case 1 had gingival hyperplasia with mucosal bleeding and friability and bilateral upper and lower extremity edema with diffuse purpura. (B) The patient in case 2 had diffuse, erythematous, papular rash over his bilateral upper and lower extremities, and on his anterior trunk. (C) The patient in case 3 had scattered purpura on bilateral upper extremities with right-side predominance and a saddle nose deformity.

A previously healthy 14-year-old male presented to an emergency department of a hospital in the southeastern United States with epistaxis. Six weeks prior to presentation, he was febrile with complaints of sore throat, epistaxis, petechial rash, headache, chest discomfort, and joint and back pain. Two weeks prior to presentation, he reported bleeding of his gums, gross hematuria, and decreased urine output. In the emergency department, his temperature was 38.4°C, pulse was 106 beats per minute, blood pressure was 178/109 mmHg, respiratory rate was 20 breaths per minute, and oxygen saturation was 95% at room air. Notably, on physical examination, the patient had gingival hyperplasia with mucosal bleeding and friability, injected conjunctiva, decreased airy entry on left lung fields, and bilateral lower extremity edema with diffuse purpura (Figure 1A). The patient also reported a recent 15lbs weight loss. He denied a history of upper respiratory or gastrointestinal symptoms, previous episodes of melena, hematochezia, or hematemesis, known sick contacts, recent travel, or trauma. The patient received vancomycin and ceftriaxone before being admitted to the pediatric intensive care unit (PICU) as sepsis was considered a differential diagnosis. 

**Figure 2:**
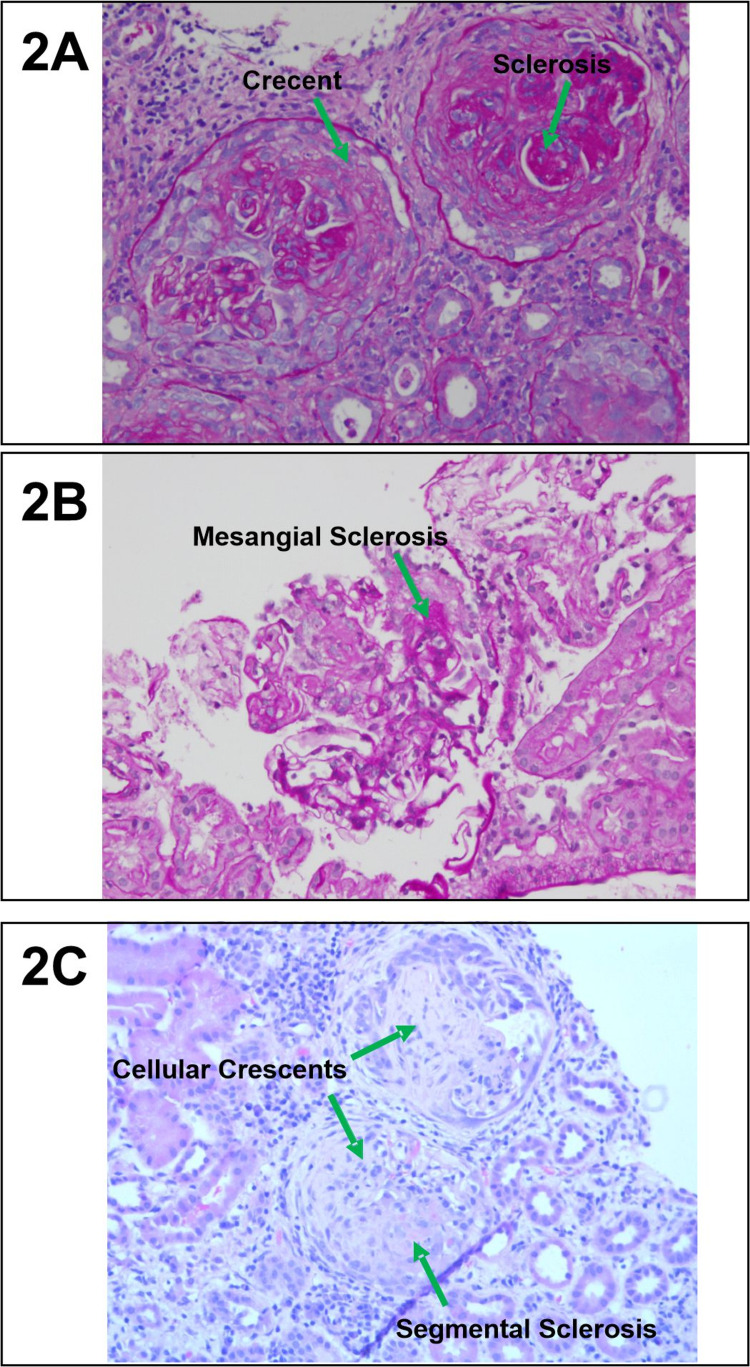
Renal biopsy findings of pauci-immune glomerulonephritis of three patients. (A) The patient in case 1 had 42% fibrocellular crescents and sclerosis. (B) The patient in case 2 had 7.7% sclerosis without the presence of crescents. (C) The patient in case 3 had 60% glomerulosclerosis (global and segmental) and 40% cellular or fibrocellular crescents.

Laboratory workup included a serum creatinine (Cr) of 1.25mg/dL (normal: 0.45-0.81mg/dL), a urinalysis (UA) significant for hematuria and proteinuria with a urine protein-to-creatinine (Up/c) ratio of 2 (normal <0.2), erythrocyte sedimentation rate (ESR) 90mm/h (normal: 0-10mm/h), and CRP 15.8mg/L (normal: 0.1-1.0mg/L), serum c-ANCA of 1:640 and proteinase-3 (PR3) of 1501U/mL (Table 1). Of note, the patient was negative for p-anti-neutrophilic cytoplasmic antibody/myeloperoxidase (ANCA/MPO), glomerular basement membrane (GBM), C3/C4, antisense oligonucleotide (ASO), and lupus anti-coagulants. Chest CT was significant for evidence of focal pulmonary hemorrhage. Renal biopsy showed 42% (20/47) of glomeruli had fibrocellular crescents on hematoxylin and eosin stain (H&E) and no deposits noted on electron microscopy (EM) - findings consistent with pauci-immune crescentic glomerulonephritis (Figure 2A).

**Table 1:** Admission data on three patients. Of note, all patients were negative for p-ANCA/MPO, GBM, C3/C4, ASO, and lupus anticoagulants. Cr: creatinine; p/c: protein-to-creatinine ratio; ESR: erythrocyte sedimentation rate; CRP: C-reactive protein; c-ANCA: cytoplasmic anti-neutrophil cytoplasmic antibody; PR-3: proteinase-3; GBM: glomerular basement membrane; ASO: antisense oligonucleotide; MPO: myeloperoxidase

	Serum Cr level (mg/dL)	Urine p/c ratio	Hematuria	ESR (mm/h)	CRP (mg/L)	c-ANCA	PR-3 (U/mL)
Case 1	1.25	2	present	90	15.8	1:640	1501
Case 2	0.6	0.79	present	20	0.28	1:80	841
Case 3	3.26	2.8	present	127	103	1:640	188.7
Reference values	0.40-1.00	< 0.2	absent	0-12	0-10	< 1:10	< 20

Given serology positive for c-ANCA/PR3, abnormal UA findings, evidence of pulmonary hemorrhage, and renal biopsy consistent with pauci-immune crescentic glomerulonephritis, the patient met the European League Against Rheumatism, Pediatric Rheumatology International Trials Organization, and Pediatric Rheumatology European Society (EULAR/PRINTO/PRES) criteria for granulomatosis with polyangiitis (GPA) [18].

The patient received induction therapy with intravenous (IV) methylprednisolone 1g x3 doses daily, oral (PO) prednisolone 60mg/d, IV cyclophosphamide (Cytoxan) 1000mg x6 doses (750mg/m2 per month), IV rituximab 375mg/m2 x2 doses, therapeutic plasma exchange (TPE) x5 rounds, and IVIG x1 round. Maintenance therapy included daily PO prednisolone with a tapering schedule and azathioprine (Table 2).

**Table 2:** Treatment of induction and maintenance for three patients. TPE: therapeutic plasma exchange; IVIG: IV immunoglobulin; ANCA: anti-neutrophil cytoplasmic antibody; PR-3: proteinase-3; ESR: erythrocyte sedimentation rate; CRP: C-reactive protein; Cr: creatinine; eGFR: estimated glomerular filtration rate; CKD: chronic kidney disease

	Induction therapy	Maintenance therapy	Relapse therapy
Case 1	IV Solu-Medrol 1g x3 doses; oral prednisolone 60 mg/d; IV cytoxan 1000mg x6 doses (750mg/m^2^ per month); IV rituximab (375mg/m^2 ^x2 doses) TPE x5 rounds; IVIG x1 round	Oral prednisone daily with tapering azathioprine	One relapse about two years after diagnosis, which was responsive to IV steroid pulse 1g x3 doses and rituximab (375mg/m^2 ^x3 doses); passed away two years after relapse episode from an unrelated illness following transition to adult nephrology care
Case 2	IV Solu-Medrol 1g x3 doses; oral prednisolone 60mg/d; IV cytoxan 1000mg x6 (750mg/m^2^ per month; total 5.9g); IV rituximab (375 mg/m^2 ^x4 doses) TPE x6 rounds; no IVIG	Oral prednisone daily with tapering azathioprine	No clinical relapse since diagnosis in 2014; one recurrence of PR-3-positive serology in January 2018, which responded well to IV rituximab; off steroid due to cushioned feature; tolerated azathioprine well; last seen in August 2018
Case 3	IV Solu-Medrol 1g x3 doses; IV Solu-Medrol 500mg x8 doses per week; oral prednisolone 50mg/d; IV cytoxan 500-850mg/m^2^ x7 doses; total 5.6g); IV rituximab (375mg/m^2 ^x4 doses); no TPE or IVIG	Oral prednisone daily with tapering azathioprine rituximab as needed	After induction therapy, repeat renal biopsy revealed 74% of glomeruli with global sclerosis, whereas no cellular crescents; labs showed negative ANCA/PR-3, ESR, and CRP with improvement; serum Cr ranges 0.71, eGFR 83mL/min/1.73m^2 ^(CKD stage II); followed up by Pediatric Nephrology and Rheumatology monthly

Remission was successfully achieved in this patient; however, approximately two years after the diagnosis was made, the patient had a relapse, which was responsive to IV steroid pulse 1g x3 doses and rituximab 375mg/m2 x3 doses per institution protocol. Two years after his episode of relapse, the patient passed away from an unknown unrelated illness following the transition to adult nephrology care (Table 2).

Case 2 (patient with diffuse, petechial rash)

A previously healthy 13-year-old male presented to an emergency department of a hospital in the southeastern United States with epistaxis, which he reports had been intermittent for the past four weeks. Six weeks prior to presentation, the patient noted joint pain with bilateral upper extremity edema. Three weeks prior to the presentation, he had a purpuric rash on his bilateral ankles. In the emergency department, he was afebrile, normotensive, and saturating adequately on room air without tachypnea or tachycardia. On physical examination, he was noted to have a diffuse, erythematous, papular rash over his bilateral upper and lower extremities, as well as across his anterior trunk. Scattered flesh-colored papules were also noted over his bilateral inner thighs (Figure 1B).

Laboratory workup included a serum Cr of 0.6mg/dL, a UA significant for hematuria and proteinuria with Up/c of 0.79, ESR 20mm/hr, and CRP 0.28mg/L. Serum c-ANCA of 1:80 and PR-3 of 841U/mL (Table 1). Of note, the patient was negative for p-ANCA/MPO, GBM, C3/C4, ASO, and lupus anti-coagulants. The renal biopsy that was performed showed: 7.7% (1/13) of glomeruli had sclerotic lesions on H&E and no deposits noted on EM - findings consistent with pauci-immune crescentic glomerulonephritis (Figure 2B).

Given serology positive for c-ANCA/PR3, abnormal UA findings, signs of upper airway involvement including epistaxis, and renal biopsy consistent with pauci-immune crescentic glomerulonephritis, the patient met the European League Against Rheumatism, Pediatric Rheumatology International Trials Organization, and Pediatric Rheumatology European Society (EULAR/PRINTO/PRES) criteria for GPA [18].

The patient received induction therapy with IV methylprednisolone 1g x3 doses, PO prednisolone 60mg/d, IV cyclophosphamide 1000mg x6 doses (750mg/m2 per month; total 6.0g), IV Rituximab 375mg/m2 x4 doses, and TPE x6rounds. IVIG was not given to this patient. Maintenance therapy included daily PO prednisolone with a tapering schedule and azathioprine (Table 2).

Since the diagnosis was made in 2014, the patient had no clinical relapse. There was one recurrence of PR3-positive serology with a decline in renal function four years after his initial presentation, which was responsive to IV Rituximab. He was deemed appropriate to completely wean off steroid therapy but continued to tolerate azathioprine well. The patient was last seen in the summer of 2018 without any known reports of relapse or other acute events (Table 2).

Case 3 (patient with persistent proteinuria, hematuria, and worsening serum creatinine)

A previously healthy 10-year-old female, who immigrated from Iraq at the age of five years, presented to an emergency department of a hospital in the northeastern United States with vomiting. One year prior to presentation, she reported epistaxis, hip pain, and abdominal pain with nausea and intermittent vomiting. Six months prior to presentation, she was seen at a local children’s hospital emergency department for hip pain with effusion, which was treated symptomatically and yielded clinical improvement. On her presentation, her temperature was 36.8°C, pulse was 89 beats per minute, blood pressure 108/57, respiratory rate was 17 breaths per minute with oxygen saturation at 100% on room air. Notably, on examination, the patient had a saddle nose deformity, scattered purpura on bilateral upper extremities with right-side predominance, and arthritis of right ankle (Figure 1C).

On admission, serum Cr was 3.26mg/dL, a UA significant for hematuria and proteinuria with Up/c of 2.8, ESR 127mm/h, and CRP 103mg/L. Serum c-ANCA of 1:80 and PR3 of 188.7U/mL (Table 1). Of note, the patient was negative for p-ANCA/MPO, GM, C3/C4, ASO, and lupus workup. Renal biopsy showed 60% glomerulosclerosis (global and segmental) and 40% cellular or fibrocellular crescents - findings consistent with pauci-immune crescentic glomerulonephritis (Figure 2C). Chest CT showed minimal patchy ground-glass opacities and two equivocal nodular densities in the lower lobe segments. Sinus CT showed prominent nasal septum perforation with mild mucosal thickening in the left maxillary antrum. Echocardiogram also performed was unremarkable.

Given serology positive for c-ANCA/PR3, abnormal UA findings, signs of upper airway involvement with sinusitis and lung involvement, and renal biopsy consistent with pauci-immune crescentic glomerulonephritis, the patient met the European League Against Rheumatism, Pediatric Rheumatology International Trials Organization, and Pediatric Rheumatology European Society (EULAR/PRINTO/PRES) criteria for GPA [18].

The patient received induction therapy with IV methylprednisolone 1g x3 doses with 500mg x8 doses per week, PO prednisone 2mg/kg daily, IV cyclophosphamide 500-850mg/m2 x7 doses (total 5.6g), and IV Rituximab 375mg/m2 x4 doses. TPE and IVIG were not given to this patient. She was also on Enalapril given her HTN and iron for anemia. Maintenance therapy included daily PO prednisone, azathioprine, and Rituximab (Table 2).

Following induction therapy, repeat renal biopsy showed 74% of glomeruli had global sclerosis with no cellular crescent was observed. Repeat labs returned with ANCA-negative and PR3-negative serologies with normalizing of ESR and CRP. With regard to renal function, repeat serum Cr of 0.7 mg/dL with an eGFR of 83 mL/min/1.73m2, which placed the patient in the category of chronic kidney disease, stage II (Table 2).

One year after diagnosis the patient presented vomiting and headaches, which progressed to double vision and left cranial nerve VI palsy. Workup did not demonstrate GPA relapse, however, the examination demonstrated bilateral papilloedema. Lumbar puncture was significant for elevated opening pressure of 36cm H20 with CSF analysis being negative for signs of the infectious process. Ultimately, the patient was diagnosed with idiopathic intracranial hypertension (IIH) and was treated with acetazolamide which was escalated from 5mg/kg to 10mg/kg throughout the course of the admission. The patient’s clinical symptoms improved and she was back at baseline by discharge. The patient continues to be followed up monthly by Pediatric Nephrology and Rheumatology to date.

## Discussion

Granulomatosis with polyangiitis (GPA) is an anti-neutrophilic cytoplasmic antibody (ANCA)-associated vasculitis (AAV) that is characterized by systemic granulomatous and necrotizing inflammation affecting the small- and medium-sized blood vessels. AAVs are autoimmune disorders in which autoantibodies are generated against neutrophil granular proteins, namely protease-3 (PR3) and myeloperoxidase (MPO). GPA is most commonly associated with PR-3 ANCAs, which leads to the characteristic clinical manifestations of the disease [19]. While GPA can involve any organ system, it most commonly affects the upper and lower respiratory tracts and the kidneys [18-23].

Childhood-onset GPA is rare and often manifests with non-specific symptoms and variations in the degree of involvement of each system at initial presentation. Ear, nose, and throat (ENT) tract manifestations are the most common features at disease presentation in childhood-onset GPA, followed by constitutional symptoms, nephropathy, and lower respiratory tract involvement [20]. The heterogeneous nature of clinical manifestations at disease onset, with a particular propensity of GPA for the upper respiratory tract, was adequately demonstrated by the cases discussed in this paper. Apart from a prolonged history of epistaxis, all the patients had remarkably different chief complaints on initial presentation.

Renal disease associated with GPA is typically characterized by rapidly progressive and crescentic glomerulonephritis, which can ultimately lead to end-stage renal disease [19,20]. Clinically, this may present with hematuria, proteinuria, and hypertension as well as elevations in creatinine on laboratory workup. The three presented patients demonstrated the variations in the severity of renal disease of GPA, with the patient in case 1 being the only one symptomatic of rapidly progressive glomerulonephritis at onset, with gross hematuria, oliguria, and hypertensive emergency. In contrast, the patients in cases 2 and 3 initially only demonstrated laboratory and histopathological evidence of renal involvement with the patient in case 2 progressing to stage II chronic kidney disease.

Regarding pulmonary involvement, GPA typically manifests with non-cavitating or cavitating pulmonary nodules on high-resolution computed tomography (HRCT) and alveolar hemorrhage [21]. While these manifestations are less common than upper respiratory tract and renal disease, the consequences are potentially life-threatening. In patients with GPA who have a significant decline in hemoglobin and new-found ground-glass opacities on high resolution computed tomography (HRCT), it is imperative that the clinician consider pulmonary hemorrhage and implement interventions as early as possible to avoid catastrophic outcomes. The patients in cases 1 and 3 had evidence of lung involvement on imaging, although did not develop symptoms of alveolar hemorrhage.

While central nervous system (CNS) involvement is not included in the “classic triad” of clinical manifestations of GPA, it occurs in approximately 10-45% of patients [22]. Typically, it presents with headaches, sensory or motor impairments, vestibular involvement, or hearing loss. It is important to consider infectious etiologies, particularly bacterial meningitis, with this clinical presentation as infectious disease is a significant source of morbidity and mortality in GPA patients. Following full work-up and effectively ruling out the infectious cause, the patient in case 3 was diagnosed with pseudo-tumor cerebri (IIH). This is the first documented case of IIH in GPA, and it is unknown whether it is specifically related to the disease process of GPA or treatment of GPA such as the steroid wean or if it is related to another confounding variable altogether [2].

Despite the different initial presentations, all three patients met the diagnostic criteria for GPA based on the pediatric-specific classification of vasculitis by EULAR/PRES [18]. As such, it is essential for clinicians to have a low index of suspicion for GPA and consider the EULAR/PRES criteria in patients with a protracted course of upper respiratory tract symptoms, particularly in the context of hematuria, proteinuria, and lung disease.

The prognosis of GPA has markedly improved with the discovery and appropriate use of immunosuppressive therapy in the past number of decades, with a five-year survival rate currently at approximately 70-80% [19]. Important prognostic factors related to childhood-onset GPA include the presence of pulmonary hemorrhage and the severity of renal disease indicated by the percentage of normal glomeruli on renal biopsy and the eGFR at diagnosis [23]. The three discussed cases are consistent with these findings. The patient in case 2 had the least significant renal disease, with normal creatinine and only 1/13 glomeruli with sclerotic lesions on renal biopsy at initial presentation. Following treatment, he remained in remission with no other complications, as compared to his counterparts in cases 1 and 3, both of whom had close to 50% of glomeruli affected with sclerosis and elevated serum creatinine at presentation and both of whom experienced more significant morbidity of GPA. Therefore, these cases further demonstrate the importance of renal biopsy, not only for diagnosis of GPA, but more importantly for prognostication and ultimately management.

## Conclusions

GPA is a serious disease, with a mortality rate that approaches 100% within one year if patients remain untreated. Despite the seriousness and potentially fatal nature of this disease, there is lack of literature regarding the approach to treating pediatric patients with GPA. As such, pediatric nephrologists and rheumatologists have had to extrapolate from adult-based clinical trials to determine the most appropriate management plan for their patients with GPA. The patients in this case series all received similar treatment with induction therapies including IV steroid pulse followed by PO steroids and cyclophosphamide for six months and rituximab. Intensive plasmapheresis was typically reserved for those patients with severe disease, including those requiring dialysis, those with pulmonary hemorrhage, and those patients who fail to respond to therapy. Maintenance therapy in these cases included PO steroids and azathioprine for two years. All three patients responded appropriately and went into remission with undetectable ANCA-antibody levels following induction. The patients in cases 1 and 3 experienced relapse and CKD, respectively, although this might have been expected given the degree of renal disease at onset. Despite these complications and vastly different initial clinical presentations, we postulate that the treatment regimen administered to these patients was appropriate and effective at inducing remission in pediatric patients with GPA.
